# With Our Editors

**Published:** 1906-10-15

**Authors:** 


### WITH OUR EDITORS.

**THE FAILURE OF CONGRESS TO PASS THE ARMY AND NAVY DENTAL BILLS.**

The interesting communication of Dr. S. D. Boak, Dental Surgeon U. S. Army published upon another page, sets forth so clearly the value of the service rendered the army by the hard working corps of civilian appointees now acting as army dental surgeons under the contract system, as to emphasize the regret which all must feel that Congress adjourned without passing the bill giving them commissioned rank, with the pay, emoluments and recognized social status accorded to all commissioned officers in the army, and that the bill providing for commissioned dental surgeons in the United States Navy also failed of enactment.

If there is small consolation in the fact that the bill for the re-organization of the medical corps of the army and the substitution of commissioned rank for the present contract surgeon system in that branch of the service was equally unsuccessful, we are at least spared the increased chagrin which would have attended the passage of the medical and the failure of the dental bills.

At this time there need be no attempt to analize or explain the causes of this failure; they were doubtless manifold. The army dental bill, as originally framed, had certain defects, which were sharply criticised, this resulting in so much contention and work at cross-purposes before Congressional committees and elsewhere, that some of the best friends of the measure, in and out of Congress, lost heart and interest in its advocacy. Independent of all this, however, a leading factor in the failure of the bill was the unrelenting opposition of an influential Senator from a New England State, from which section greater enlightenment and a wiser judgment was to have been expected.

Much of the criticism of the bill, as originally framed by the committee, the *Brief* regarded as unwise because endangering its passage. Probably, with or without criticism or amendment, the result would have been the same; but as the perfect bill has yet to be framed, it is, on general principles, usually best in Congressional legislation to let a measure well advanced in its passage go through, provided it fairly well meets the essential needs, for which it was framed and trust to future legislation to correct defects and secure betterments.

Both for the army dental surgeon and the dental profession at large, the most vital point in the bill was that it secured commissioned status for the dental corps, and this on conditions which were so fairly adequate as to justify their acceptance, subject to a wise administrative interpretation of terms and details.

When, however, later in the session a member of the House Committee having the bill in charge embodied therein an amendment expressly designed to make Drs. Marshall Oliver and other faithful members of the army dental corps, on a question of age limit at the time of their original appointment, ineligible for further service, the *Brief* entered a protest against the injustice of such a measure.

In this connection it may be mentioned that Dr. Emory A. Bryant, editor of *The National Dental Critic*, while not at all disapproving of the editorial, takes exception to what he regards as the inconsistency of the editor, Dr. Bryant having been advised by letter, in February last, that, for reasons practically the same as those above stated, it was deemed inexpedient to publish, at that time, in the *Brief*

his criticism of the then pending army dental bill and of the Committee on Army and Navy Dental Legislation of the National Dental Association.

As the editor of the *Brief* understands the situation, however, the conditions were vastly different in February, 1906, when the letter to Dr. Bryant was written, and in June, 1906, when the editorial on "A Proposed Injustice to Many Members of the Army Dental Corps" appeared; the editorial contained no criticism of the committee or of the bill as originally framed, nor was it written at a time when there was a hopeful outlook for its success. In any case, it is better to be right than to be consistent, and at no time would the *Brief* have been willing to accept a bill containing so obnoxious a provision as that against which its protest was entered.

The one important lesson taught by the failure of the army and navy dental bills is that legislative battles are to be won, not by divided counsels, but by unity of purpose and harmony of action in the conduct of the compaign. Legislation to secure adequate dental service for the army and navy is of too much importance, both to the defensive forces of the nation and to dentistry as a profession, to be allowed to suffer defeat because of individual differences and grievances among those who should be united in the common purpose to secure the best possible enactment on the best possible terms.

The National Dental Association, at its coming session, should look well to this question, which is by far the most important it will have under consideration. Not partisanship, but wise statesmanship is needed. Let there be enlisted in the cause all who are able to do effective work in securing legislative support.

The failures of the past are but incidents in a movement, the ultimate success of which is certain. In such a case a temporary check is not final failure; rather is it renewed opportunity for the still better organization and prosecution of a campaign of education, a campaign already so far advanced, both in the Senate and House of Representatives, there is probably a clear working majority won over to the conviction that skilled dentistry for the army and navy is an imperative need which must be met. The only points seriously at issue are statutory details regarding organization upon which there are still conflicting views. Concerning these, the vital features to be insisted upon are that whatever dental acts are passed shall meet in some adequate degree the present needs of the army and navy, and at the same time give just recognition to dentistry as a profession and just consideration to its representatives now in military service.—Dr. W. F. Litch, in September *Brief*.

**OUR CREED.**

"Trade is occupation for livelihood;
Profession is occupation for service of the world.
Trade is occupation for joy of the result;
Profession is occupation for joy in the process.
Trade is occupation where anybody may enter;
Profession is occupation where only those who are prepared may enter.
Trade is occupation taken up temporarily until something better offers.
Profession is occupation with which one is identified for life.
Trade makes one the rival of any other trader;
Profession makes one the co-operator with all his colleagues.
Trade knows only the ethics of success;
Profession is bound by lasting ties of sacred honor."

*Pres. Faunce of Brown Univ., before R. I. Med. Soc.*

This wise and thoughtful statement of President Faunce goes so deeply into the root of the matter that we place it here as a motto up to which we are trying to live.

We believe that dentistry is really a profession, not a trade; and we find this belief borne out by a careful examination of this statement. But it is never wise in beginning a new work to ignore good work that has already been done in a similar direction. Hence, we have no quarrel with the various journals which are controlled by and represent various trade interests; they are honest and true to their purpose. They have great excellence in their own direction and are wisely calculated to aid, not only their supporting trade, but also those persons upon whose trading they depend. In furtherance of this end they spend money freely, employing the ablest men they can find who will accept their service, and make costly use of all the arts of printing and illustration. This is honest, straightforward and true, and the men who serve them faithfully are worthy of honor. The excellencies of these journals are so well and so fully appreciated, that it is not necessary to dwell further upon them. But if it is true that dentistry is a profession and not a mere trade, then it is surely time that the profession as a whole should be bound together by one or more publications which should represent and be controlled by the profession itself, and not by a trade which lives upon it. If the profession is really an oak, it should bear its own fruit and not allow a parasite, drawing its life from the tree, to put forth its fruit as the fruit of the tree. The oak should bear acorns which have the seed of life in them, and not merely mistletoe berries, which, however beautiful, can never grow oaks. And why do we want acorns? Because we want oaks. For the profession needs its own publication, not only as its proper fruit, to show to the world that it really lives, but also as the seed from which may spring a new and vigorous growth.

At bottom the profession is a thought—a thought of service of the world, and such a thought needs expression not only in service, but in words which may quicken to life in other minds the desire to serve. Who may speak such words for a true profession? Can those whose real purpose is not to serve but to sell, who, in spite of all excellence, are frankly and openly traders?

We believe that we are a true profession; that our fundamental thought is really and truly a desire to serve our fellows. And we are founding this journal that not only we ourselves may better serve our fellows, but that we may help to deepen in the minds of others the thought of service to the world. We seek also the binding force of a common purpose, that controlling the expression of our own thought of service is not hidden by the thought of gain, for the natural selfishness of man needs every help, if it is to be changed to unselfishness.

The vision that comes before us is that of all members of the dental profession bound together in a great band, even as their brothers of the medical profession are bound. Each seeking to perfect himself in his ability to serve and to aid in the perfecting of every one else. Each one striving that the thought of service may have the final triumph. That as we have freely received, so we may freely give.—Dr. Charles A. Kimball, editorial in the July *Journal*, the new organ of the New York Institute of Stomatology and other Societies.

**A PHASE OF ETHICS.** It would sometime seem as if dentists overlooked a very important professional obligation in their treatment of the patients of other practitioners who chance to fall into their hands temporarily in an emergency. The code of ethics is somewhat specific as to what is a man's duty under such circumstances, but there are too many in the profession who are either ignorant of the code or else wittingly violate it.

When a patient who is regularly the patron of one dentist is obliged to go to another in some emergency, such, for instance, as a lost filling, a broken bit of enamel, or the toothache, while the family dentist is absent from his office, or while the patient is traveling, the duty of the dentist thus applied to is self-evident. He should relieve the temporary disability so that the patient is protected from injury or discomfort, and then refer the patient back to the regular dentist. This seems so plain, so equitable and so in keeping with all that is ethical and honest, that no one need do what is wrong through ignorance.

And yet it is too often the case that the dentist, when appealed to for temporary relief, does not scruple to take advantage of the patient's disability and insinuatingly search out all the ills, real or imaginary, which he can find in the mouth, and then proceed, if the patient will allow him, to do the most expensive work he can.

The really unfortunate part of this is not the financial loss to the regular dentist, but the creation of a suspicion in the mind of the patient that the dentist has been lax in his attention to the teeth and has overlooked much necessary work. Not only this, but frequently great injury is done the patient in the performance of work which the judgment of the regular practitioner, based on a long observation of the patient's teeth, would have guarded against. No one knows so well what is best in a given case as the man who has conscientiously studied the mouth for a number of years, and it is frequently a case of "fools rushing in where angels fear to tread."

A typical instance of this was related to the writer by a careful and painstaking practitioner, who is noted for his vital interest in his patient's welfare. A gentleman had a lower incisor, which for many years had been somewhat loose, but had been retained with comfort to the patient by a little attention during occasional periods of soreness. On one such occasions the patient was away from home and applied to another dentist for temporary relief, reciting to him the history of the case. The new dentist assured the patient that there was no need of the occasional disturbances, that the case could be readily cured permanently, and finally persuaded him to have the tooth extracted, replanted and banded to the adjacent tooth. The patient returned to his regular dentist in six weeks to have the band removed, under the impression that a permanent cure had been effected, and with the intimation that he regretted this had not been done before. The regular practitioner said nothing about the advisability of the operation, but suggested that the band be allowed to remain a little longer. After a few more weeks the patient insisted on the removal of the band, saying that the tooth was perfectly firm and comfortable. In a very short time the tooth began to drift out of place and had to be banded again. In less than a year it was lost, despite the most strenuous effort to save it.

The operator who replanted it probably did so with the best intentions, but there is said to be a certain very undesirable place that is paved with good intentions, and under the circumstances the dentist who was applied to for temporary relief exceeded his duty in such a radical procedure.

It is, of course, true that no dentist should see a patient suffering from manifestly wrong treatment on the part of the regular practitioner without taking the means of remedying the difficulty, but this is a diffierent question from the one we are considering. Probably the most prevalent abuse is the tendency to crown teeth for transient patients without warrant. It so frequently happens that teeth which have been serviceably preserved by filling for years by the regular dentist, and which might be saved for years to come with a slight repairing of the filling, are ruthlessly slashed off and crowned the moment the patient falls temporarily into the hands of another. This kind of treatment is not only wrong in practice, but wrong in principle, and it has grown to such proportions as to constitute a serious reflection on the integrity and good judgment of many in the profession. It would be well if some of our practitioners were to study more carefully their true function toward transient patients, and live up to the spirit of professional and fraternal equity and justice in their treatment of them.—C. N. Johnson, in October *Review*.

**THE DUTIES OF STATE BOARDS OF EXAMINERS.** Along with the state laws passed in the past few years for the regulation of the practice of the various professions, laws intended to protect the people from the abuses of the unscrupulous and the quacks, come numerous complaints against state boards, not alone against state boards of dental examiners, but against other state boards as well.

One of the most recent cases which has come to our notice is that against the State Board of Pharmacy of the State of Maryland. It seems that a chemist made application for a license to practice as a drug clerk in Baltimore, and the Board of Pharmacy refused to grant an examination on the grounds that the applicant was not a graduate of a school of Pharmacy. The applicant, believing he was entitled to an examination, took the matter into the courts, and as a result secured, at the hands of the Supreme Court of the United States, a ruling to the effect that it is not within the province of a state board, appointed by the governor, to inquire either *how, when* or *where* a person has attained proficiency to practice his calling, but rather to inquire *has* he acquired such proficiency, and, further, that it is the duty of the board to give every applicant a chance to demonstrate his fitness.

This ruling of the Supreme Court is of interest to the dental profession, for what applies to the Board of Pharmacy of the State of Maryland will apply to all state boards of all states, and, we take it, that it will therefore be necessary to amend many of the laws, which, as they now exist, are really inoperative and unconstitutional.

In many states the state board of dental examiners recognize a diploma issued by a reputable school of dentistry as the necessary qualification, either for the issuance of a license to practice, or for the privilege of taking an examination, but according to this recent decision we believe it will devolve upon the state boards to go further and give an examination to any applicant, whether or not he be the holder of a diploma, and find out regarding his ability to properly serve those entrused to his care.

Not infrequently is heard complaint from some applicant for a license that he has not received just and proper treatment at the hands of the state board of dental examiners. It is claimed, and we have seen in print intimations coming from some members of state boards, that it is the duty of the state boards of dental examiners to protect the dental profession from overcrowding, but such a holding is plainly illegal and not well founded, and what must be done in Maryland must be done in other states.

It would seem at the present time, notwithstanding the vast improvement made in the last few years, both in the dental laws and in the personnel of the board of examiners, that there is yet much improvement and change to be made. Whether or not the faults lie in the laws, in the boards or in both, is an open question. The real duty of a state board of dental examiners is, according to our idea, to protect the people from abuse and injury at the hands of the incompetent.

Some change can be brought about by the National Association of Dental Faculties, made up of representatives of the recognized schools. Their rules governing requirements, graduation, etc., are uniform. and let them do away with the rule that says a student *must* be in attendance three or four years and substitute a more just rule that will allow a student to take an examination for advanced standing and go ahead if he be qualified and has the skill. One student may be able to do in one year what another will do in two years, and it is manifestly unfair for the diligent and talented of a class to be held back by the slothful and stupid. If a student can, after two years' schooling, acquire the necessary knowledge and skill to practice in his profession, he should not be compelled to spend another year in school.

It would be absurbed in this day and age to take the position that it is not of *very great* advantage for a student to enter one of the well-equipped dental schools to get his training, but if he sees fit to get his training outside the dental school, according to the ruling of the court, he is entitled to an examination and credentials if he can show that he is qualified.

The National Association of Dental Examiners should prepare for a discussion of these questions, as they may come up for consideration and adjustment. Though, under the court ruling, state boards cannot raise a question as to schooling or credentials, the boards will be the most important factors in the professions. The responsibility of the boards will be increased and it is important that they get together and, as far as possible, unify not only the laws and rules, but examinations as well, so that a man who has obtained a license in any state may be allowed to practice in any other state.

When such a conditions obtains, friction and strife will be banished and equity and justice established.—J. N. Crouse, in July *Digest.*

**NATIONAL DENTAL ASSOCIATION MEETING AT ATLANTA.**

The Atlanta Meeting of this body was a crisis in its history and the profession will be pleased to learn that it has passed leaving the Association with a brighter prospect than ever before.

The work of the sections was more satisfactory than at any previous meeting, due to the fact that each section in turn was given the use of the main meeting-hall, so that there was no conflict of programs. Thus authors met larger audiences and the discussions were freer and fuller. Some very good papers were offered and the literary part of the work may be said to have been an advance over other years.

But the chief good was attained behind closed doors, and will not become fully apparent except as the months roll by, and it be discovered that the warring factions in the National have really buried their personal differences, and will adhere to the compromises agreed upon. If this be done, and all members hereafter work in harmonious unison, there is every prospect that the Association may become National in character as well as in name.

There is no disguising the fact that a number of men journeyed to Atlanta with bitterness in their hearts and a determination to have themselves and their personal views upheld at all costs. The chief matter of dispute fell under the supervision of the Executive Committee, and it is not too much to say that the members of the Executive Committee martyred themselves in an effort to terminate the disputes, to advoid public scandals, and withal to restore harmony, while at the same time leaving each contestant his self-respect and a feeling that impartial justice had been administered.

In all cases the disputants were made to see that they must accept compromise or be prepared for rigid discipline, should further investigation show that it might be merited. But above all an appeal was made, that private grievances should give away, in order that the good name of dentistry should not be smirched by scandals.

The members of the Eexcutive Committee so devoted themselves to this herculean task, that whole nights were spent in reading documents, and whole sessions given over to hearing evidence. In the end they succeeded in every instance in finding for all a compromise which could be accepted with honor. And so, if the pacified but remain pacific, and all pledges be kept, a season of harmony and united effort is drawning for the National Dental Association. And if this dawn should develop into a brighter day, it must never be forgotten that the clouds were swept away at Atlanta by the splendid zeal of the men in the Executive Committee.

Without detracting an iota from the value of the services rendered by the Executive Committee, there unquestionably was a subtle force at work which largely contributed towards the outcome, and the one word which symbolizes that force in all its manifold expressions is the word "Fraternity."

Two college Fraternities were largely represented, while nearly a hundred men wore the pin of the Interstate Dental Fraternity. This body was especially organized only four years ago with the chief purpose of disseminating a broader feeling of sociability and brotherly love at the annual meetings of the National Dental Association. The Asheville, St. Louis and Buffalo meetings were all very successful in in promoting this object, but the Atlanta banquet was singularly potent in its immediate effect.

When the members sat down to the dinner, no sort of settlement of the secret strifes had been found, nor seemed possible, and a suppressed restlessness impregnated the air.

But the urgent appeals of the evening's speakers for fraternal feeling, and fraternal action; for suppression of self, and forgiveness of wrongs, touched the hearts of all, and compromise immediately became possible; nay was assured. The psychological moment was reached when the last speaker, Dr. Hetrick, with impressive oratory preached optimistically of the future, and asked that all gods should be forgotten, and that all should soar with him into the higher ethers where there is but one God over all; that God which breathes only universal love. As the members rose and united in softly singing "Auld Lang Syne," all felt the inspiration of a presence, which softened and united all hearts, and those who had not known him best, and missed him most, guessed that the departed Chappelle was with us in spirit.

May harmony and brotherly love continue to prevail and may the future usefulness of the National Dental Association increase with passing years.—Dr. Ottolengui, in October, *Items of Interest.*

